# Crystallographic Characterization on Polycrystalline Ni-Mn-Ga Alloys with Strong Preferred Orientation

**DOI:** 10.3390/ma10050463

**Published:** 2017-04-27

**Authors:** Zongbin Li, Bo Yang, Naifu Zou, Yudong Zhang, Claude Esling, Weimin Gan, Xiang Zhao, Liang Zuo

**Affiliations:** 1 Key Laboratory for Anisotropy and Texture of Materials (Ministry of Education), School of Material Science and Engineering, Northeastern University, Shenyang 110819, China; yangb@atm.neu.edu.cn (B.Y.); neuznf@126.com (N.Z.); zhaox@mail.neu.edu.cn (X.Z.); lzuo@mail.neu.edu.cn (L.Z.); 2 Laboratoire d’Étude des Microstructures et de Mécanique des Matériaux (LEM3), CNRS UMR 7239, Université de Lorraine, 57045 Metz, France; claude.esling@univ-lorraine.fr; 3 Laboratory of Excellence on Design of Alloy Metals for low-mAss Structures (DAMAS), Université de Lorraine, 57045 Metz, France; 4 German Engineering Materials Science Centre (GEMS), Helmholtz-Zentrum Geesthacht (HZG) Outstation at FRM II, D-85748 Garching, Germany; weimin.gan@hzg.de; 5 Taiyuan University of Science and Technology, Taiyuan 030024, China

**Keywords:** ferromagnetic shape memory alloys, texture analysis, orientation distribution

## Abstract

Heusler type Ni-Mn-Ga ferromagnetic shape memory alloys can demonstrate excellent magnetic shape memory effect in single crystals. However, such effect in polycrystalline alloys is greatly weakened due to the random distribution of crystallographic orientation. Microstructure optimization and texture control are of great significance and challenge to improve the functional behaviors of polycrystalline alloys. In this paper, we summarize our recent progress on the microstructure control in polycrystalline Ni-Mn-Ga alloys in the form of bulk alloys, melt-spun ribbons and thin films, based on the detailed crystallographic characterizations through neutron diffraction, X-ray diffraction and electron backscatter diffraction. The presented results are expected to offer some guidelines for the microstructure modification and functional performance control of ferromagnetic shape memory alloys.

## 1. Introduction

Conventional shape memory alloys (SMAs) can generate large output strains as a result of reversible martensitic transformation. However, a major inconvenience for the practical application of SMAs is their low working frequency (less than 1 Hz), since thermal activation is necessary. Recently, a significant breakthrough in the research of high performance SMAs came about with the discovery of ferromagnetic shape memory alloys (FSMAs) [1], where the magnitude of output strain is comparative to that in conventional shape memory alloys [2,3,4,5,6,7,8]. Moreover, the possibility of controlling the shape change by the application of magnetic field enables relatively higher working frequency (KHz) than that of conventional shape memory alloys. With the integration of large output and fast dynamic response under the external magnetic field, FSMAs are conceived as the promising candidates for a new class of actuation and sensing applications.

Among FSMAs, Heusler type Ni-Mn-Ga alloys are the most representative prototype [9,10], which combine the properties of ferromagnetism with those of a reversible martensitic transformation. Due to the strong coupling between the magnetic and structural orders, these alloys can demonstrate giant output strains under the actuation of the magnetic field through the reorientation of ferromagnetic martensite variants [1,2,3,4,5,6,7,8,9,10,11,12,13,14], i.e., magnetic shape memory effect. On cooling, Ni-Mn-Ga alloys undergo the martensitic transformation from austenite with cubic L2_1_ structure to the low symmetry martensitic phase with several possible structures. Depending on the composition [15], the product phase could be five-layered modulated (5M), seven-layered modulated (7M) and non-modulated (NM) martensite [16,17,18,19,20,21,22,23,24,25]. Among them, the NM martensite has a simple tetragonal crystal structure [25], whereas the 5M and 7M martensite possess the monoclinic superstructure with lattice modulation that is reflected by the satellite spots between two main spots in electron diffraction patterns [16,17,18,19,20,21,22,23,24]. In addition to the martensitic transformation, there also exists a first-order intermartensitic transformation from one type of martensite to another in some alloys [26,27,28,29,30,31].

During the last two decades, numerous experimental studies have been conducted on the composition-dependent magnetic shape memory behavior in Ni-Mn-Ga alloys. Thus far, the field induced output strains have almost reached the theoretical limit in single crystals, i.e., ~7%, ~11% and ~12% in single crystals with 5M, 7M and NM martensite [6,7,8], respectively. It should be noted that the high-cost for the fabrication of single crystals represents a severe obstacle for practical applications. In contrast, the preparation of polycrystalline alloys are much simpler and of lower cost. However, a more or less random distribution of crystallographic orientation in polycrystalline alloys greatly weakens the field controlled functional behavior. To improve the functional properties in polycrystalline alloys, microstructure optimization and texture control are of great significance and challenge. In this paper, we present our recent progress on the microstructure control in polycrystalline Ni-Mn-Ga alloys in the form of bulk alloys, melt-spun ribbons and thin films, based on the detailed crystallographic characterizations through neutron diffraction, X-ray diffraction and electron backscatter diffraction (EBSD). For the bulk alloys, which were prepared by directional solidification, the thermo-mechanical treatment (compressive load applied during the martensitic transformation) was introduced in order to redistribute the variants. For the melt-spun ribbons with strong preferential orientation, the orientation inheritance between austenite and 7M martensite was analyzed. For the thin films deposited on MgO(1 0 0) substrate, the preferential orientation and variant distribution were illustrated.

## 2. Experimental

Bulk polycrystalline alloys with the nominal composition of Ni_50_Mn_30_Ga_20_ (at. %) and Ni_50_Mn_28.5_Ga_21.5_ (at. %) were prepared by directional solidification. In order to obtain a composition homogenization, the directionally solidified Ni_50_Mn_30_Ga_20_ and Ni_50_Mn_28.5_Ga_21.5_ bulk alloys were homogenized at 1173 K for 24 h in a sealed vacuum quartz tube, followed by the quenching into water. A part of homogenized alloy was ground into powder and then the powder was annealed at 873 K for 5 h in vacuum to release the internal stress for the subsequent powder X-ray diffraction (XRD) measurements.

Ribbons with the nominal composition of Ni_53_Mn_22_Ga_25_ (at. %) and Ni_51_Mn_27_Ga_22_ (at. %) were produced through single-roller melt-spinning with a Cu wheel rotating speed of 15 m/s. Thin films with nominal composition of Ni_50_Mn_30_Ga_20_ (at. %) and nominal thickness of 1.5 μm were deposited from a cathode target of Ni_46_Mn_32_Ga_22_ (at. %) by DC magnetron sputtering with a sputtering rate of ~0.2 nm/s. A Cr buffer layer of 100 nm thick was pre-coated on the MgO(1 0 0) monocrystal substrate.

The room-temperature crystal structure was determined by X-ray diffraction (XRD) with Cu-K*α* radiation. The martensitic transformation temperatures were measured by differential scanning calorimetry (DSC, TA Q100) with a heating and cooling rate of 10 K/min. The microstructural characterization was performed in a field emission gun scanning electron microscope (SEM, Jeol JSM 6500 F) with an EBSD acquisition camera and Channel 5 software. 

**Figure 1:**
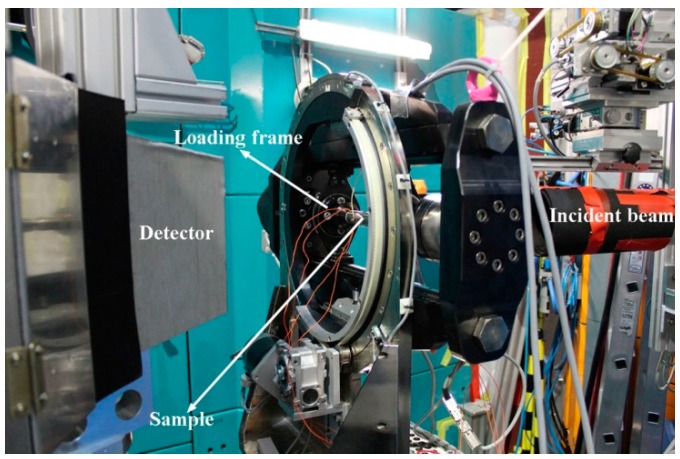
The unique tensile/compression rig installed at STRESS-SPEC for in-situ neutron diffraction measurements.

The neutron diffraction measurements were performed using the materials science diffractometer STRESS-SPEC operated by FRM II and HZG at the Heinz Maier-Leibnitz Zentrum (MLZ), Garching, Germany, with a monochromatic wavelength of 2.1 Å [32]. The uniaxial compressive load was applied by a rotatable multifunctional (tension/compression/torsion) load frame installed at STRESS-SPEC [33], with the “constant load” mode to ensure a fixed load. The experimental setup for in-situ neutron diffraction is shown in Figure 1. For the in-situ neutron measurements on the directionally solidified alloys during thermo-mechanical treatment process, the sample was firstly heated to austenite temperature region, where a certain uniaxial compressive load was applied along the solidification direction. Then, the sample was cooled to room temperature at a cooling rate of 2 K/min under the constant load, during which the neutron diffraction patterns were continuously recorded at an interval of 60 s by a two-dimensional (2D) detector. During the in-situ neutron diffraction measurements, the macroscopic strain changes of the tested samples were measured by the clip-on extensometers in the load frame. Besides, the global crystallographic textures of both the initial sample (without thermo-mechanical treatment) and the sample after thermo-mechanical treatment were examined by neutron diffraction. The incoming beam sizes for the in-situ neutron diffraction and the pole figure measurements were 5 mm×5 mm and *φ*15 mm, respectively. 

## 3. Results and Discussion

### 3.1. Thermo-Mechanical Treatment of Directionally Solidified Alloys

In general, the martensitic transformation is deformation-dominant diffusionless phase transformation with symmetry break. The lower symmetry of the product martensitic phase may result in the formation of self-accommodated multi-variants to compensate the elastic strains associated with the phase transformation. However, such self-accommodated microstructure is not favorable for the achievement of magnetic shape memory effect in Ni-Mn-Ga alloys, since the co-existence of multi-variants would greatly enhance the resistance for the variant reorientation. As the deformation accompanying the martensitic transformation is anisotropic, unidirectional constraint (tension or compression) during the martensitic transformation could promote the formation of certain favorable variants but eliminate some other unfavorable ones [34]. Therefore, strong preferential orientation of martensite can be achieved through the selective formation of favorable variants with the application of an external field during the martensitic transformation, thus to realize the optimization of crystallographic anisotropy and magnetic shape memory effect [2,4,13,14,35]. In this section, thermo-mechanical treatments (compressive load applied during the martensitic transformation) were introduced in order to reformulate the variant distribution. Through in-situ neutron diffraction, the martensitic transformation process under uniaxial compressive load was traced and the direct evidence on the variant redistribution induced by thermo-mechanical treatments was followed.

#### 3.1.1. Austenite to 7M Martensite Transformation

Polycrystalline Ni_50_Mn_30_Ga_20_ alloy with 7M martensite at room temperature was prepared by directional solidification. The cylindrical-shaped (*φ*5 mm×10 mm) samples with the axial direction parallel to solidification direction were cut from the homogenized ingot for thermo-mechanic treatment and neutron diffraction. The actual composition was verified to be Ni_50.1_Mn_28.8_Ga_21.1_ by energy dispersive spectroscopy (EDS). According to DSC measurements, the start and finish temperatures of the forward (*M_s_*, *M_f_*) and inverse martensitic transformation (*A_s_*, *A_f_*) were determined to be 347.8 K, 331.3 K, 336.8 K and 352.2 K, respectively. Powder XRD measurements reveal that the directionally solidified Ni_50_Mn_30_Ga_20_ alloy consists of 7M martensite at room temperature with lattice parameters *a_7M_* = 4.2651 Å, *b_7M_* = 5.5114 Å, *c_7M_* = 42.365 Å, and *β* = 93.27°, where the crystal structure of 7M martensite is depicted as an incommensurate monoclinic superstructure consisting of ten unit cells [22]. Moreover, microstructural observations have shown that the original austenite of the directionally solidified alloy forms coarse columnar-shaped grains with the grain size of several hundreds of microns along the solidification direction (SD) [36]. 

**Figure 2:**
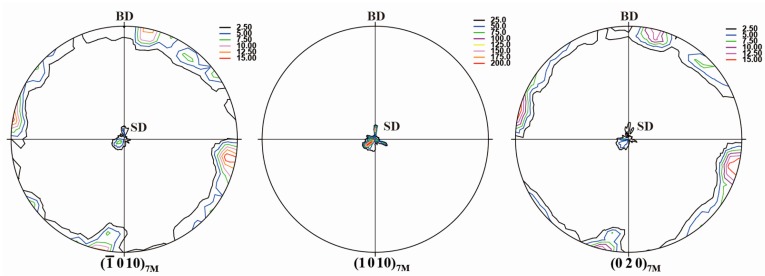
{−1 0 10}_7M_, {1 0 10}_7M_ and {0 2 0}_7M_ complete pole figures of 7M martensite measured from neutron diffraction for the directionally solidified Ni_50_Mn_30_Ga_20_ alloy (BD: incoming beam direction; SD: solidification direction).

To reveal the global texture of the directionally solidified Ni_50_Mn_30_Ga_20_ alloy, the complete pole figures were measured by neutron diffraction. The high penetration capability of neutrons, which exceeds that of X-rays by about four orders of magnitude, is considered to allow more reliable analysis on the global orientation distribution of the studied samples. Figure 2 displays {−1 0 10}_7M_, {1 0 10}_7M_ and {0 2 0}_7M_ complete pole figures of the directionally solidified alloy. It is seen that the 7M martensite develops a strong preferential orientation, with {−1 0 10}_7M_, {1 0 10}_7M_ and {0 2 0}_7M_ crystallographic planes of 7M martensite either perpendicular or parallel to the SD. For {−1 0 10}_7M_ and {0 2 0}_7M_, the orientation component parallel to the SD possesses the much higher intensity, indicating that {−1 0 10}_7M_ and {0 2 0}_7M_ tend to be parallel to SD. On the other hand, {1 0 10}_7M_ is almost perpendicular to the SD. According to the orientation relationship between austenite and 7M martensite [37], {−1 0 10}_7M_, {1 0 10}_7M_ and {0 2 0}_7M_ of 7M martensite are originated from {2 0 0}_A_ of austenite. Thus, it can be inferred that the initial austenite of the directionally solidified Ni_50_Mn_30_Ga_20_ alloy should possess the strong <0 0 1>_A_ preferential orientation parallel to the solidification direction.

**Figure 3:**
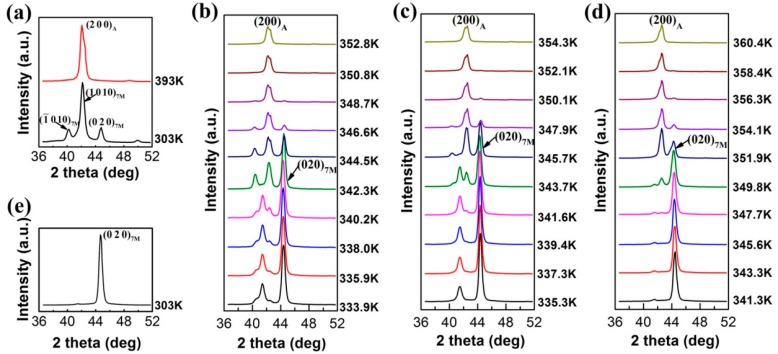
(**a**) Neutron diffraction pattern measured at ~393 K and ~303 K with no external load; (**b**–**d**) neutron diffraction patterns measured during cooling under the compressive load of −10 MPa (Cycle 1), −25 MPa (Cycle 2) and −50 MPa (Cycle 3); and (**e**) neutron diffraction pattern measured at ~303 K without compressive load after three cycles of treatment [36].

In order to modify the variant distribution, cyclic thermo-mechanical treatments were performed on the directionally solidified Ni_50_Mn_30_Ga_20_ alloy, and the martensitic transformation process under external load was traced by in-situ neutron diffraction. The sample was first heated to 393 K to reach the fully austenite state, where the uniaxial compressive load was applied along the solidification direction (SD). Since the austenite of the directionally solidified Ni_50_Mn_30_Ga_20_ alloy possesses the strong <0 0 1>_A_ preferential orientation in parallel to the solidification direction, the uniaxial compressive load was actually applied along the <0 0 1>_A_. Prior to thermo-mechanical treatment, the neutron diffraction patterns were collected at 393 K and 303 K in the 2*θ* range of ~36°–52° for the tested sample, as shown in Figure 3a. It is seen that within measured 2*θ* range, only {2 0 0}_A_ diffraction can be observed in the austenite (*a_A_* = 5.83 Å) temperature region. After martensitic transformation, {2 0 0}_A_ evolves into {−1 0 10}_7M_, {1 0 10}_7M_ and {0 2 0}_7M_, where the {1 0 10}_7M_ diffraction possesses the strongest intensity. 

Figure 3b–d displays the serial patterns measured on cooling across the martensitic transformation under the compressive load of −10 MPa (Cycle 1), −25 MPa (Cycle 2) and −50 MPa (Cycle 3) applied along the solidification direction, respectively. With the increase of compressive load, the intensity of {0 2 0}_7M_ diffraction increases gradually. After three cycles of treatment, there remained almost only the {0 2 0}_7M_ diffraction in the measured 2*θ* range, as shown in Figure 3e. Apparently, the uniaxial compression has exerted significant influence on the variant distribution, creating a strong preferential orientation of the {0 2 0}_7M_. Moreover, with increasing the compressive load, the macroscopic deformation amount accompanying the martensitic transformation increased gradually, i.e., −2.1%, −2.8% and −3.3% for Cycle 1, Cycle 2 and Cycle 3, respectively, which also indicates the increase in the degree of preferred variant orientation.

The applied uniaxial compressive load can also result in the increase of martensitic transformation temperatures. Experimentally, the increases of *M_s_* under −10 MPa, −25 MPa and −50 MPa applied during martensitic transformation were ~0.9 K, ~2.3 K and ~8.5 K, respectively [36]. The shifts of transformation temperatures under uniaxial load *σ* can be well explained by the Clausius–Clapeyron relation: d*σ*/d*T* = −∆*S*·*ρ*/*ε*, where ∆*S* and *ε* stand, respectively, for the entropy change and transformation strain, and *ρ* is the mass density. According to Clausius–Clapeyron relation, the increase of *M_s_* under uniaxial load of −10 MPa, −25 MPa and −50 MPa was determined as 1.2 K, 4.1 K and 9.5 K, respectively, which is very close to the experimentally observed transformation temperature shifts [36].

**Figure 4:**
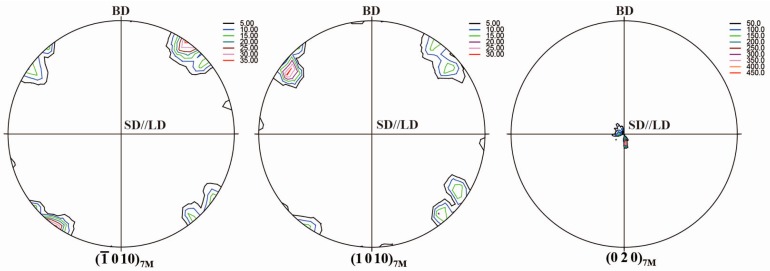
{−1 0 10}_7M_, {1 0 10}_7M_ and {0 2 0}_7M_ pole figures of 7M martensite after cyclic thermo-mechanical treatments measured from neutron diffraction for the directionally solidified Ni_50_Mn_30_Ga_20_ alloy (BD: incoming beam direction; SD: solidification direction; LD: loading direction).

Figure 4 displays {−1 0 10}_7M_, {1 0 10}_7M_ and {0 2 0}_7M_ pole figures of 7M martensite after cyclic thermo-mechanical treatments. Compared to the initial sample without thermo-mechanical treatment (Figure 2), a significant change on the crystallographic orientation of 7M martensite can be confirmed. It is seen that {−1 0 10}_7M_ and {1 0 10}_7M_ of 7M martensite are almost parallel to the loading direction (LD) (also the SD), whereas {0 2 0}_7M_ is almost perpendicular to LD. Notably, a strong <0 1 0>_7M_ preferential orientation along the LD was induced by the external compression during martensitic transformation. With respect to {2 0 0}_A_ (d = 2.915 Å), the plane spacing of resultant {−1 0 10}_7M_ (d = 3.09 Å) and {1 0 10}_7M_ (d = 2.919 Å) increases and {0 2 0}_7M_ (d = 2.756 Å) decreases after martensitic transformation. Under the compressive load applied during martensitic transformation, the variants with the reduction in the plane spacing should be more favorable. Thus, the formation of {0 2 0}_7M_ from {2 0 0}_A_ is preferred under compressive load, leading to the large macroscopic strain and the formation of a strong <0 1 0>_7M_ preferred crystallographic orientation along the loading axis.

#### 3.1.2. Austenite to 5M Martensite Transformation

In order to figure out the effect of external loading direction applied during the martensitic transformation on the selection of preferential variants, a directionally solidified Ni_50_Mn_28.5_Ga_21.5_ alloy with two preferred orientations with <0 0 1>_A_ and <1 1 0>_A_ parallel to the solidification direction (SD) was used to perform thermo-mechanical treatments. The rectangular parallelepiped samples (10 mm × 6.5 mm × 6.5 mm) with their longitudinal direction parallel to the solidification direction were cut from the homogenized alloy for neutron diffraction testing. 

According to the EDS, the actual composition was determined to be Ni_49.6_Mn_28.4_Ga_22.0_. Powder XRD measurement shows that the alloy composes of 5M martensite at room temperature and the lattice parameters were determined to be *a_5M_* = 4.226 Å, *b_5M_* = 5.581 Å, *c_5M_* = 21.052 Å and *β* = 90.3°. DSC measurements demonstrate that the martensitic transformation occurs above the room temperature. The start and finish temperatures of the forward and reverse martensitic transformation determined from DSC measurements are, respectively, 322.9 K (*M_s_*), 318.4 K (*M_f_*), 329.5 K (*A_s_*) and 333.2 K (*A_f_*) [38].

**Figure 5:**
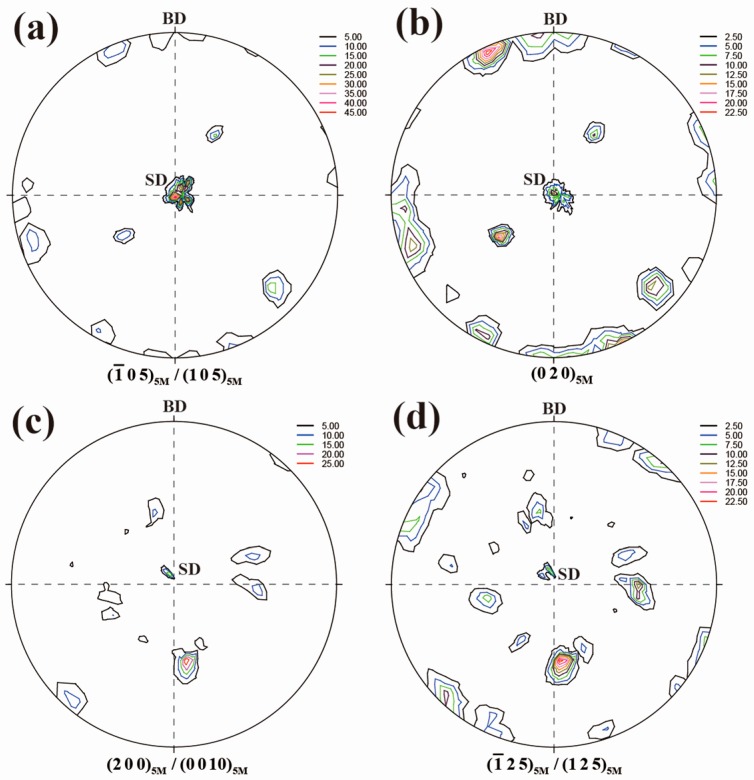
Complete pole figures of directionally solidified Ni_50_Mn_28.5_Ga_21.5_ alloy without thermo-mechanical treatment measured from neutron diffraction (BD: incoming beam direction; SD: solidification direction) [38]: (**a**) {1 0 5}_5M_/{−1 0 5}_5M_ pole figure; (**b**) {0 2 0}_5M_ pole figure; (**c**) {0 0 10}_5M_/{2 0 0}_5M_ pole figure; and (**d**) {1 2 5}_5M_/{−1 2 5}_5M_ pole figure.

Figure 5 shows the {1 0 5}_5M_/{−1 0 5}_5M_, {0 2 0}_5M_, {2 0 0}_5M_/{0 0 10}_5M_ and {1 2 5}_5M_/{−1 2 5}_5M_ complete pole figures measured from neutron diffraction for the directionally solidified Ni_50_Mn_28.5_Ga_21.5_ alloy. It is noted that the angular differences in 2*θ* between (1 0 5)_5M_ and (−1 0 5)_5M_, between (2 0 0)_5M_ and (0 0 10)_5M_ and between (−1 2 5)_5M_ and (1 2 5)_5M_ are too small, i.e., ~0.2°, ~0.3° and ~0.2°, respectively, to be effectively distinguished in the present neutron diffraction measurements due to the instrumental resolution of the diffractometer, resulting in the overlapping of two diffractions with very close 2*θ*, i.e., (1 0 5)_5M_ and (−1 0 5)_5M_, (2 0 0)_5M_ and (0 0 10)_5M_, and (−1 2 5)_5M_ and (1 2 5)_5M_, in the neutron diffraction measurement results, respectively [38]. 

It is seen in Figure 5 that both the {1 0 5}_5M_/{−1 0 5}_5M_ and {0 2 0}_5M_ poles are roughly located at the tilt angle Psi = ~0°, ~40° and ~90° in the corresponding pole figures. Since {1 0 5}_5M_/{−1 0 5}_5M_ and {0 2 0}_5M_ of 5M martensite are originated from {2 0 0}_A_ for the transformation from austenite to 5M martensite [39], it can be inferred that the initial austenite of directionally solidified Ni_50_Mn_28.5_Ga_21.5_ alloy mainly possesses two preferred orientation components, i.e., <0 0 1>_A_//SD and <1 1 0>_A_//SD. Thus, during the subsequent thermo-mechanical treatment process, the compressive load applied along SD during the martensitic transformation can be viewed along <0 0 1>_A_ and <1 1 0>_A_. For the initial austenite with the preferred orientation of <0 0 1>_A_//SD, the resultant {1 0 5}_5M_/{−1 0 5}_5M_ and {0 2 0}_5M_ of martensite are either perpendicular or parallel to the SD. More specifically, for {1 0 5}_5M_/{−1 0 5}_5M_, the orientation component perpendicular to the SD possesses the much higher intensity, indicating that {1 0 5}_5M_/{−1 0 5}_5M_ tends to be perpendicular to SD. On the other hand, for {0 2 0}_5M_, the orientation component parallel to the SD has the higher intensity. For the initial austenite with the orientation of <1 1 0>_A_//SD, the resultant {2 0 0}_5M_/{0 0 10}_5M_ tends to be perpendicular to SD and {1 2 5}_5M_/{−1 2 5}_5M_ to be parallel to SD.

**Figure 6:**
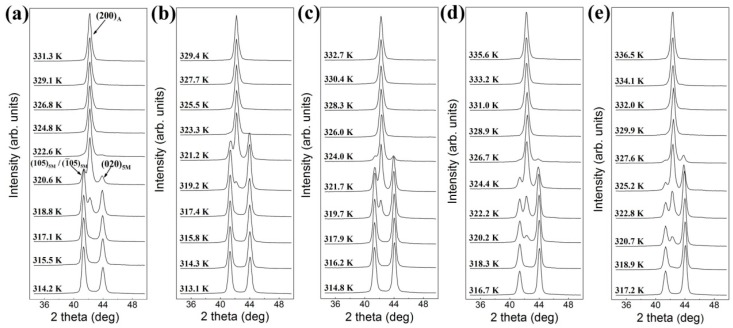
In-situ neutron diffraction patterns measured on cooling under the compressive load for directionally solidified Ni_50_Mn_28.5_Ga_21.5_ alloy [38]: (**a**) 0 MPa (Cycle 1); (**b**) −10 MPa (Cycle 2); (**c**) −20 MPa (Cycle 3); (**d**) −40 MPa (Cycle 4); and (**e**) −50 MPa (Cycle 5).

Figure 6a shows the in-situ neutron diffraction patterns measured on cooling without external load (Cycle 1). Within the measured 2*θ* range (~36°–48°), only the {2 0 0}_A_ diffraction can be observed in the austenite temperature region. The lattice constant of the austenite was determined to be *a_A_* = 5.84 Å. On cooling, {2 0 0}_A_ transforms into {1 0 5}_5M_/{−1 0 5}_5M_ and {0 2 0}_5M_, where {1 0 5}_5M_/{−1 0 5}_5M_ diffraction possesses a higher intensity than {0 2 0}_5M_ diffraction. Figure 6b–e displays the serial patterns measured on cooling across the martensitic transformation under the compressive load of −10 MPa (Cycle 2), −20 MPa (Cycle 3), −40 MPa (Cycle 4) and −50 MPa (Cycle 5), respectively. For each cycle of the thermo-mechanical treatment, there remained two diffractions of 5M martensite, i.e., {0 2 0}_5M_ and {1 0 5}_5M_/{−1 0 5}_5M_, in the measured 2*θ* range after the martensitic transformation. However, the intensity ratio between {0 2 0}_5M_ and {1 0 5}_5M_/{−1 0 5}_5M_ increases with the increase of compressive load, suggesting a redistribution of martensitic variants induced by the compressive load applied during the martensitic transformation [38].

**Figure 7:**
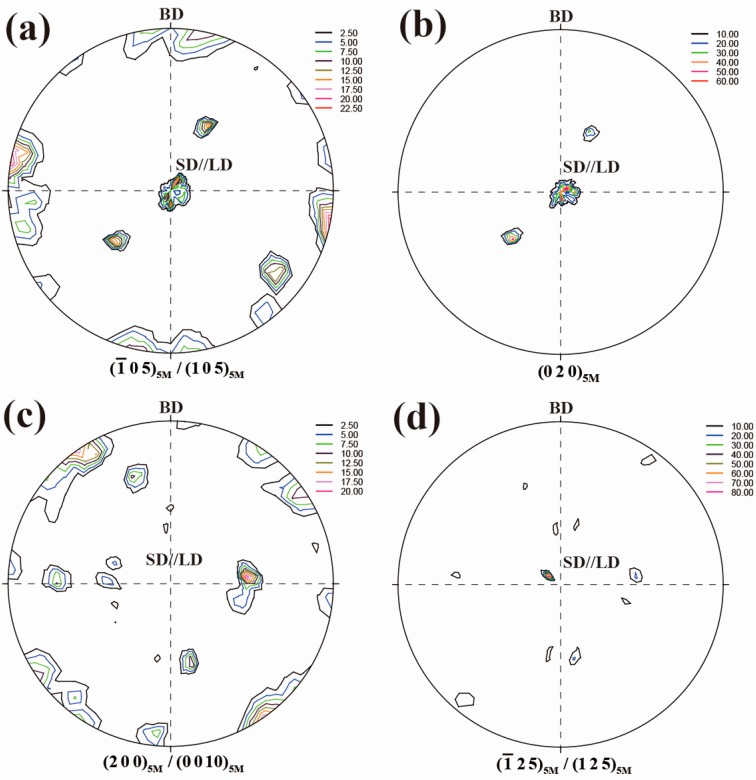
Pole figures of directionally solidified Ni_50_Mn_28.5_Ga_21.5_ alloy after five cycles of thermo-mechanical treatment: (**a**) {1 0 5}_5M_/{−1 0 5}_5M_ pole figure; (**b**) {0 2 0}_5M_ pole figure; (**c**) {0 0 10}_5M_/{2 0 0}_5M_ pole figure; and (**d**) {1 2 5}_5M_/{−1 2 5}_5M_ pole figure (BD: incoming beam direction; SD: solidification direction; LD: loading direction) [38].

Figure 7 shows the corresponding pole figures measured by neutron diffraction after five cycles of thermo-mechanical treatment for directionally solidified Ni_50_Mn_28.5_Ga_21.5_ alloy. It should be mentioned that, although the pole figures presented in Figure 5 (without thermo-mechanical treatment) and Figure 7 (after thermo-mechanical) were obtained from two different samples, the initial texture of two samples should be very similar since they were cut from the same directionally solidified alloy rod and one sample was just adjacent to the other when cutting. It is seen that under the compressive load applied along <0 0 1>_A_ during the martensitic transformation (Figure 7a,b), the preferential variants with {1 0 5}_5M_/{−1 0 5}_5M_//SD (LD) and {0 2 0}_5M_⊥SD (LD) were induced, in contrast with the initial state with {1 0 5}_5M_/{−1 0 5}_5M_⊥SD and {0 2 0}_5M_//SD. Under the compressive load applied along <1 1 0>_A_ (Figure 7c,d), the initial orientation component with {2 0 0}_5M_/{0 0 10}_5M_⊥SD and {1 2 5}_5M_/{−1 2 5}_5M_//SD evolves into {2 0 0}_5M_/{0 0 10}_5M_//SD (//LD) and {1 2 5}_5M_/{−1 2 5}_5M_⊥SD (LD) [38]. Thus, the variant orientation distribution under the compressive load applied during the martensitic transformation is strongly dependent on the austenite orientation and the direction of the external load.

With respect to {2 0 0}_A_ (d = 2.92 Å), the planar spacing of inherited {1 0 5}_5M_/{−1 0 5}_5M_ (d = 2.975 Å/2.991 Å) increases, whereas {0 2 0}_5M_ (d = 2.791 Å) decreases. Similarly, the planar spacing of {2 0 0}_5M_/{0 0 10}_5M_ (d = 2.105 Å/2.113 Å) increases, but {1 2 5}_5M_/{−1 2 5}_5M_ (d = 2.035 Å/2.040 Å) decreases in comparison with {2 2 0}_A_ (d = 2.065 Å). Under the constraint of compressive load along SD during martensitic transformation, the formation of {0 2 0}_5M_⊥SD from {2 0 0}_A_ of austenite and {1 2 5}_5M_/{−1 2 5}_5M_⊥SD from {2 2 0}_A_ of austenite should be preferentially activated to accommodate the external constraint. Therefore, the coupling between anisotropic lattice distortion in martensitic transformation and the external constraint dominates the preferred orientation of the martensite variants formed under the external constraint applied during the martensitic transformation [38].

### 3.2. Orientation Inheritance from Austenite to 7M Martensite in Melt-Spun Ribbons

The rapid solidification based on melt-spinning technique has been proven to be an effective processing route for the preparation of ribbon shaped ferromagnetic shape memory alloys [40,41,42,43,44,45,46,47,48,49,50]. This method can avoid long time post heat treatment to achieve the composition homogeneity. Moreover, melt-spun ribbons usually tend to form a highly textured microstructure [51]. In this section, Ni_53_Mn_22_Ga_25_ and Ni_51_Mn_27_Ga_22_ ribbons with austenite and 7M martensite at room temperature respectively, were prepared by melt-spinning. The preferred orientation of austenite and 7M martensite in ribbons was presented and their correlation was further analyzed.

DSC measurements show that the martensitic transformation temperatures of Ni_53_Mn_22_Ga_25_ (*M_s_* = 290 K, *M_f_* = 276 K, *A_s_* = 286 K, *A_f_* = 298 K) and Ni_51_Mn_27_Ga_22_ (*M_s_* = 323 K, *M_f_* = 308 K, *A_s_* = 318 K, *A_f_* = 331 K) ribbons are below and above room temperature, respectively. According to the XRD measurements on the ribbon plane, the room temperature phase of Ni_53_Mn_22_Ga_25_ ribbons is determined to be austenite with cubic L2_1_ structure (*a_A_* = 5.814 Å), whereas Ni_51_Mn_27_Ga_22_ ribbons consist of 7M martensite (*a*_7*M*_ = 4.235 Å, *b*_7*M*_ = 5.552 Å, *c*_7*M*_ = 42.061 Å, *β* = 92.5°) at the room temperature.

**Figure 8:**
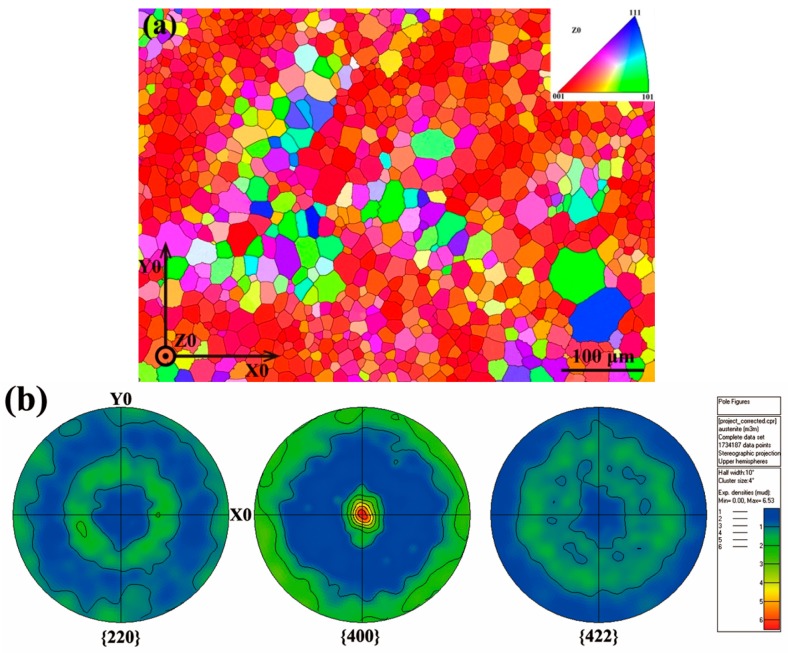
(**a**) Electron backscatter diffraction (EBSD) orientation map of ribbon plane for the Ni_53_Mn_22_Ga_25_ ribbons; and (**b**) corresponding {2 2 0}_A_, {4 0 0}_A_ and {4 2 2}_A_ pole figures. X0//ribbon length (rolling) direction, Z0⊥ribbon plane [52].

**Figure 9:**
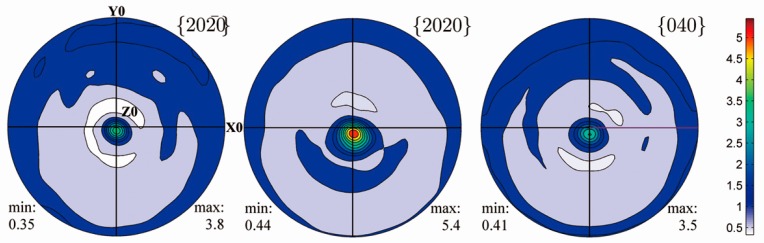
{2 0 −20}_7M_, {2 0 20}_7M_, and {0 4 0}_7M_ pole figures recalculated from the X-ray diffraction (XRD) measurements for Ni_51_Mn_27_Ga_22_ ribbons [52]. X0//ribbon length (rolling) direction, Z0⊥ribbon plane.

Figure 8a shows the EBSD orientation map measured from ribbon plane for the Ni_53_Mn_22_Ga_25_ ribbons. It is seen that the austenite grain appears in equiaxed shape in the ribbon plane with an averaged grain size of ~10–20 μm. Figure 8b displays the corresponding {2 2 0}_A_, {4 0 0}_A_ and {4 2 2}_A_ pole figures recalculated from EBSD measurements. Obviously, the austenite in ribbons develops a strong preferred orientation with {4 0 0}_A_ parallel to ribbon plane [52], which should be attributed to the thermal gradient during the melt-spinning process. Figure 9 shows the {2 0 −20}_7M_, {2 0 20}_7M_, and {0 4 0}_7M_ pole figures of 7M martensite for Ni_51_Mn_27_Ga_22_ ribbons recalculated from the XRD measurements. It is shown that the 7M martensite forms strong preferred orientation with {2 0 −20}_7M_, {2 0 20}_7M_, and {0 4 0}_7M_ parallel to the ribbon plane [52].

According to the orientation relationship between austenite and 7M martensite [37], i.e., {1 0 1}_A_//{1 −2 −10}_7M_ and <1 0 −1>_A_//<−10 −10 1>_7M_, the crystallographic correlation between austenite and 7M martensite can be well constructed. Further crystallographic calculations show that there exists an intimate correlation between {4 0 0}_A_ and its resultant {2 0 −20}_7M_, {2 0 20}_7M_, and {0 4 0}_7M_. Theoretically, {2 0 −20}_7M_, {2 0 20}_7M_, and {0 4 0}_7M_ poles of 7M martensite are almost located at the same positions with those of {4 0 0}_A_ in the corresponding pole figures calculated through the orientation relationship between two phases [52]. Based on the pole figures presented in Figure 8 (austenite) and Figure 9 (7M martensite) in ribbons, it can be inferred that the transformation from austenite to 7M martensite exhibits a strong orientation inheritance and such orientation inheritance should be attributed to the intrinsic orientation relationship between austenite and 7M martensite [52].

### 3.3. Preferential Orientation and Variant Distribution of Thin Film

The magnetron sputtering technique has been viewed as an effective method for the texturation of ferromagnetic Ni-Mn-Ga thin films epitaxially grown on a single crystal substrate [53,54,55,56,57,58,59,60,61]. In general, the epitaxial growth of Ni-Mn-Ga thin films on single crystal substrate may produce quite different microstructures compared to those of polycrystalline bulk alloys [18,20,27]. The microstructural and crystallographic characterizations of thin films remain challenging due to the local constraints from substrates, the specific geometry of thin films, and the ultrafine microstructures of constituent phases. Because of the lack of direct correlation of martensitic microstructures with crystallographic orientations, precise information on the configurations of variants in Ni-Mn-Ga thin films are still not available. In this section, based on XRD measurements and electron backscatter diffraction (EBSD) analyses, the crystal structures of constituent phases, the configurations of martensite variants and their orientation correlations are addressed.

#### 3.3.1. Global Microstructure and Texture of Thin Film

**Figure 10:**
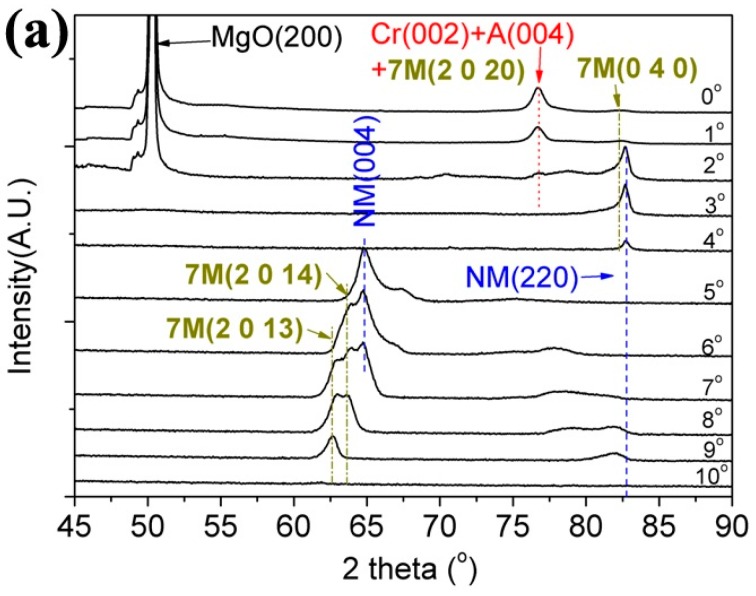

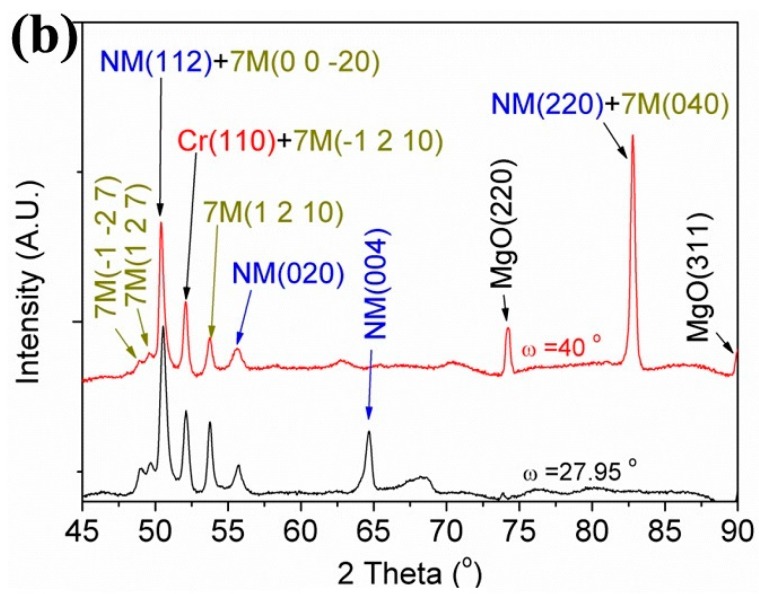
XRD patterns of as-deposited Ni_50_Mn_30_Ga_20_ thin films: (**a**) conventional *θ*–2*θ* coupled scanning at different tilt angles *ψ*; and (**b**) 2*θ* scanning at two incident angles *ω* and integrated over the rotation angle *ϕ*.

Epitaxially grown thin films with nominal composition of Ni_50_Mn_30_Ga_20_ were prepared on the MgO(1 0 0) substrate with a Cr buffer layer by DC magnetron sputtering [62,63]. Figure 10a shows the *ψ*-dependent XRD patterns of the thin films obtained by conventional *θ*-2*θ* coupled scanning at the room temperature. At each tilt angle *ψ*, there appear only a limited number of diffraction peaks. Figure 10b presents the XRD patterns measured using a large-angle position sensitive detector under two different incident beam conditions. Some extra diffraction peaks can be seen in the 2*θ* range of 48°–55°and ~82°. Based on the XRD patterns in Figure 10a,b, it can be inferred that austenite, 7M martensite and NM martensite co-exist in the as-deposited thin films at room temperature. The austenite phase has a cubic L2_1_ crystal structure with lattice constant *a_A_* = 5.773 Å. The 7M martensite phase has a monoclinic crystal structure with lattice constants *a_7M_* = 4.262 Å, *b_7M_* = 5.442 Å, *c_7M_* = 41.997 Å, and *β* = 93.7°. The NM martensite phase is of tetragonal crystal structure with lattice constants *a*_NM_ = 3.835 Å and *c*_NM_ = 6.680 Å [62,63].

**Figure 11:**
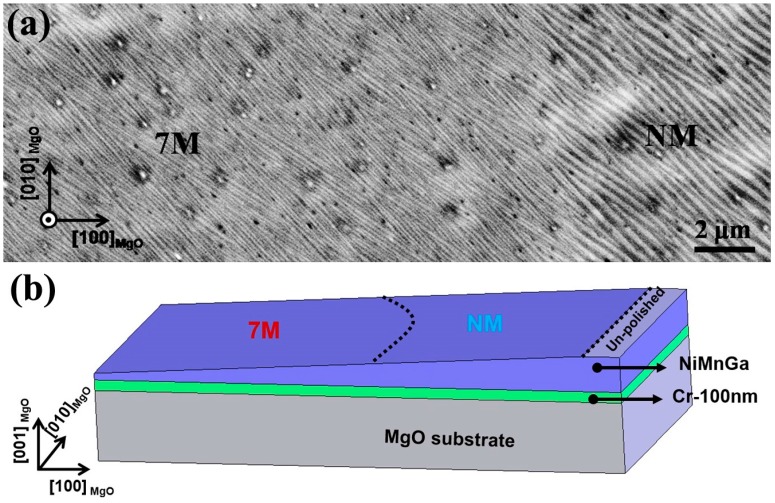
(**a**) Microstructure of electrolytically polished Ni_50_Mn_30_Ga_20_ thin film with gradient thickness relative to the film surface; and (**b**) schematic illustration of the sample thickness change from the left side to the right side [62].

Figure 11a presents a secondary electron (SE) image acquired from the top surface of an electrolytically polished sample with gradient thickness relative to the film surface. As schematically illustrated in Figure 11b, the right side and the left side of the image represent the microstructure near the film surface and deep inside the film, respectively. Although the thin film has an overall plate-like microstructure, there exists certain plate thickening from its interior to its surface, which indicates a complete change of microstructural constituents or phases along the film thickness [28]. Based on EBSD measurements [62], it is revealed that the coarse plates in the top layer of the film are of the NM martensite, whereas the fine plates in the film interior are of the 7M martensite. Moreover, by combination of the XRD results, it is deduced that the NM martensite is located near the free surface of the film, the austenite above the substrate surface, and the 7M martensite in the intermediate layers between them.

**Figure 12:**
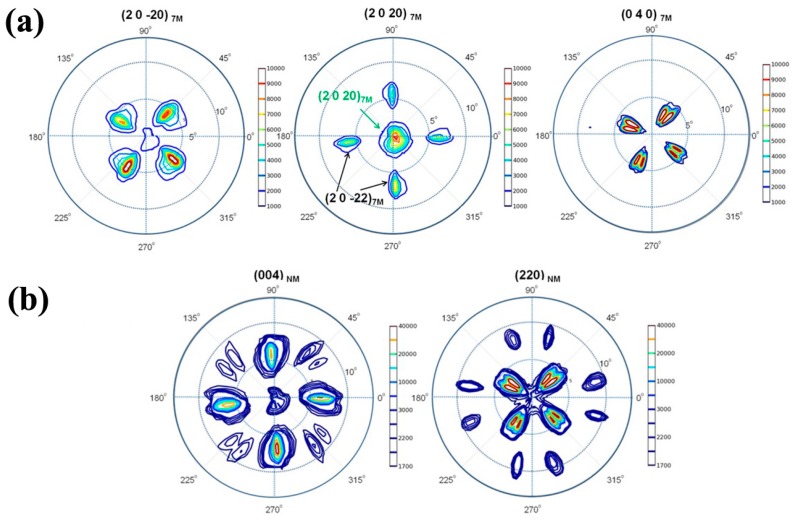
Pole figures determined from XRD measurements for 7M and NM martensite in Ni_50_Mn_30_Ga_20_ thin film: (**a**) {2 0 −20}_7M_, {2 0 20}_7M_ and {0 4 0}_7M_ pole figures; and (**b**) {0 0 4}_NM_ and {2 2 0}_NM_ pole figures.

Figure 12 displays the pole figures of the 7M and NM martensite determined from XRD measurements in the thin film. For 7M martensite (Figure 12a), {2 0 −20}_7M_, {2 0 20}_7M_ and {0 4 0}_7M_ are nearly parallel to the substrate surface, whereas for the NM martensite (Figure 12b), {0 0 4}_NM_ and {2 2 0}_NM_ tends to be close to the substrate surface. Although X-ray diffraction offers global texture information of the film, it is difficult to correlate the crystallographic features with those of the microstructure. Therefore, SEM/EBSD analysis is needed.

#### 3.3.2. 7M Variants Distribution in the Thin Film

**Figure 13:**
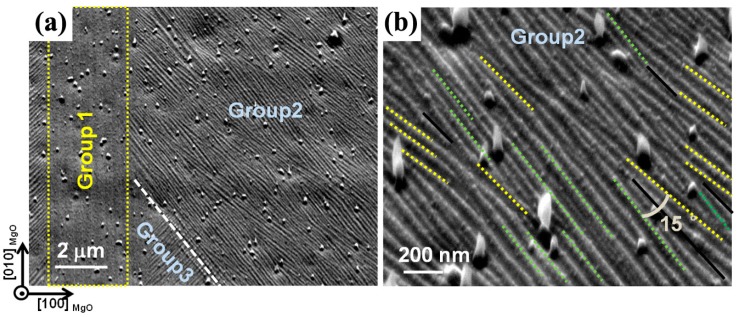
(**a**) Microstructure of 7M martensite with low relative contrast (Group 1) and high relative contrast (Group 2 and Group 3); and (**b**) magnified image of individual plates belonging to Group 2.

Figure 13 presents typical SE images of 7M martensite for Ni_50_Mn_30_Ga_20_ thin film. It is seen in Figure 13a that martensite plates are clustered in groups, exhibiting either low relative contrast (e.g., Group 1) with straight plates parallel to the substrate edges or high relative contrast (e.g., Group 2 and Group 3) with bent plates oriented at roughly 45° with respect to the substrate edges. In addition, the traces of inter-plate interfaces in high relative contrast zones have three distinct orientations, as indicated by the dotted yellow and green lines and the solid black lines in Figure 13b. In fact, the SE image contrast is related to the surface topography of an observed object. Thus, the low relative contrast zones and the high relative contrast zones are expected to have low and high surface reliefs, respectively. Here, the low relative contrast zone (Group 1) corresponds to the so-called Y pattern, and the high relative contrast zone (Group 2 or Group 3) to the X pattern [64].

EBSD measurements show that one 7M martensite plate corresponds to one orientation variant. There are in total four different variants distributed in one plate group. Here, the four orientation variants, representing one plate group with low and high relative contrast, are denoted by the symbols $V_{A}^{L}$, $V_{B}^{L}$, $V_{C}^{L}$, $V_{D}^{L}$ and $V_{A}^{H}$, $V_{B}^{H}$, $V_{C}^{H}$, and $V_{D}^{H}$, respectively. Crystallographic calculations show that there exist three types of twinning relation between the adjacent variant, i.e., Type-I, or Type-II, or compound twinning relation, for the four variants in one plate group. The complete twinning elements were reported in elsewhere [62].

**Figure 14:**
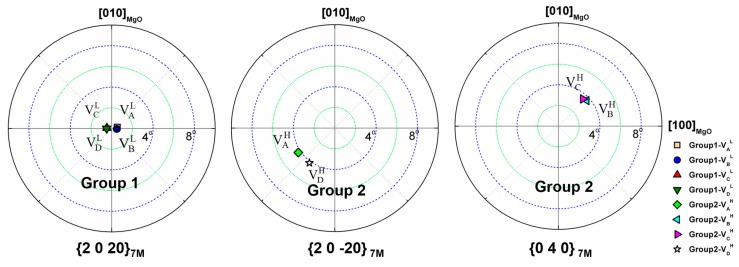
{2 0 20}_7M_, {2 0 −20}_7M_ and {0 4 0}_7M_ pole figures of four 7M variants in Group 1 and Group 2.

Figure 14 presents {2 0 20}_7M_, {2 0 −20}_7M_ and {0 4 0}_7M_ pole figures calculated from the individual crystallographic orientation data of 7M variants in Group 1 and Group 2 by manual EBSD measurements. Clearly, in the low relative contrast zone (Group 1), the four variants ($V_{A}^{L}$, $V_{B}^{L}$, $V_{C}^{L}$ and $V_{D}^{L}$) are all with their {2 0 20}_7M_ plane nearly parallel to the substrate surface. However, in the high relative contrast zone (Group 2), two variants ($V_{A}^{H}$ and $V_{D}^{H}$) are with their {2 0 −20}_7M_ plane nearly parallel to the substrate surface, and the other two variants ($V_{B}^{H}$ and $V_{C}^{H}$) with their {0 4 0}_7M_ plane nearly parallel to the substrate surface.

Analyses show that, in the low relative contrast zone, the majority of variants are in Type-I twin relation. Both Type-I and Type-II twin interfaces are nearly perpendicular to the substrate surface. For the high relative contrast zone, the majority of variants are in Type-II twin relation. The existence of height differences between adjacent variants accounts for the high relative contrast in this region. Further crystallographic calculations indicate that the preferential occurrence of different twinning type is a consequence of external constraint from the rigid substrate. The dominated twinning type allows effective cancellation of the shear deformation in the film normal direction [62].

#### 3.3.3. NM Variants Distribution in the Thin Film

**Figure 15:**
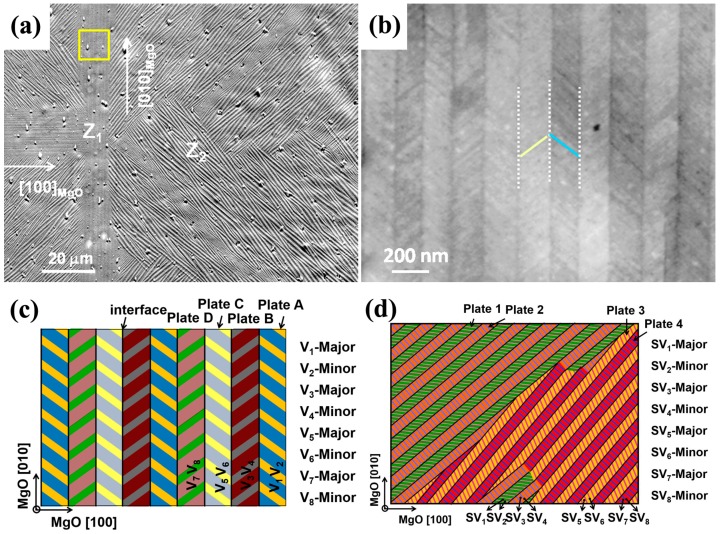
(**a**) SE image of electrolytically polished Ni-Mn-Ga thin films, showing NM martensite plates that are clustered in colonies with low (Z_1_) and high (Z_2_) relative contrasts; (**b**) high-magnification BSE image of the squared area in Z_1_ of Figure 15a, showing fine lamellae distributed alternately inside each plate; (**c**) illustration of the geometrical configuration of NM variants in Z_1_ zone; and (**d**) illustration of the geometrical configuration of NM variants in Z_2_ zone [63].

Figure 15a shows an SE image of NM martensite for Ni_50_Mn_30_Ga_20_ thin film. Similar to 7M martensite, the clustered colonies can also be characterized by two different relative contrasts, i.e., low relative contrast (Z_1_) or high relative contrast (Z_2_), as illustrated in Figure 15a. The low relative contrast zones consist of long and straight plates running with their length direction parallel to one edge of the substrate (i.e., [1 0 0]_MgO_ or [0 1 0]_MgO_). The high relative contrast zones are of shorter and somewhat bent plates that orient roughly at 45° with respect to the substrate edges.

Microstructural observation reveals that there exist two variants distributed alternately in one martensite plate, as highlighted with yellow and blue lines in Figure 15b. Of the two contrasted neighboring lamellae, one is thicker and the other is thinner, which is different from the situation of 7M martensite. The two lamellar variants in one plate have a compound twin relationship with the {1 1 2}_NM_ as twinning plane and <1 1 −1>_NM_ as twinning direction. As the BSE image contrast for a monophase microstructure with homogenous chemical composition originates from the orientation differences of the microstructural components, the thicker and thinner lamellae distributed alternately in each plate should be correlated with two distinct orientations, which is also confirmed by the indexation of Kikuchi line patterns [63].

**Figure 16:**
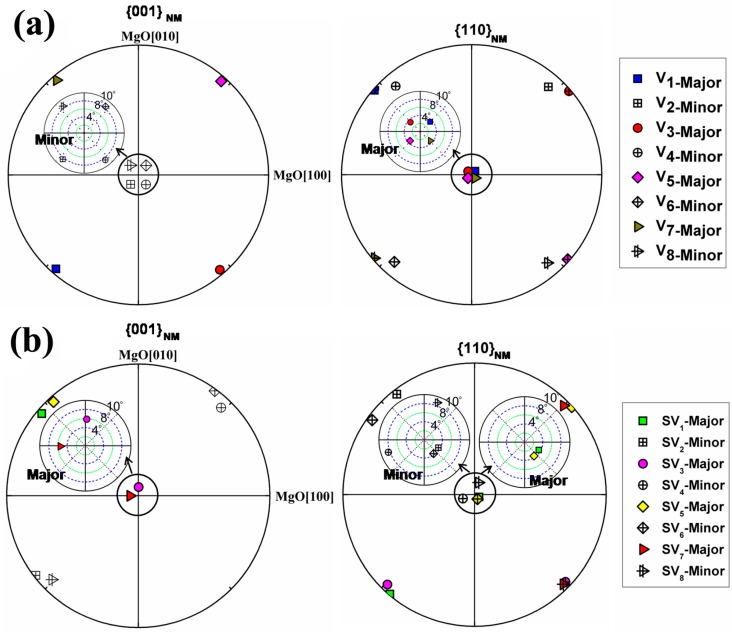
(**a**) {0 0 1}_NM_ and {1 1 0}_NM_ pole figures of NM variants in Z_1_ zone; and (**b**) {0 0 1}_NM_ and {1 1 0}_NM_ pole figures of NM variants in Z_2_ zone.

Detailed EBSD orientation analyses were conducted on the NM martensite plates in the low and high relative contrast zones (Z_1_ and Z_2_ in Figure 15a). In each variant colony, there are four types of plates, i.e., A, B, C and D in the low relative contrast (Z_1_) zones and 1, 2, 3 and 4 in the high relative contrast (Z_2_) zones, as illustrated in Figure 15c,d. Since one NM plate contains two variants, there are in total eight NM variants in one variant colony. For easy visualization, they are denoted as V_1_, V_2_, …, V_8_ in Figure 15c and SV_1_, SV_2_, …, SV_8_ in Figure 15d, where the symbols with odd subscripts correspond to the thicker (major) variants and those with even subscripts the thinner (minor) variants. The measured orientations of the NM variants in the two relative contrast zones are presented in the form of {0 0 1}_NM_ and {1 1 0}_NM_ pole figures, as displayed in Figure 16a,b [63].

For the low relative contrast zones (Z_1_), the major and minor variants are oriented respectively with their {1 1 0}_NM_ planes and {0 0 1}_NM_ planes nearly parallel to the substrate surface (Figure 16a). In the high relative contrast zones (Z_2_), such plane parallelisms hold for plates 2 and 4 but with an exchange of the planes between the major and minor variants, whereas both major and minor variants in plates 1 and 3 are oriented with their {1 1 0}_NM_ planes nearly parallel to the substrate surface (Figure 16b). In correlation with the microstructural observations, plates 2 and 4 are featured with higher brightness and plates 1 and 3 with lower brightness [63].

Indeed, for the two distinct relative contrast zones, the crystallographic orientations of the in-plate martensitic variants with respect to the substrate surface are not the same, which should be the origin of the topological differences observed for the two relative contrast zones. In the low relative contrast zones, the in-plate major and minor variants have the same orientation combination for all NM plates and they are distributed symmetrically to the inter-plate interfaces. As no microscopic height misfits across inter-plate interfaces appear in the film normal direction, the relative contrast between adjacent NM plates is not pronounced in the SE images. However, in the high relative contrast zone, the asymmetrically distributed lamellar variants in adjacent NM plates lead to the pronounced height misfits across inter-plate interfaces in the film normal direction, which gives rise to surface reliefs, hence the high relative contrast between adjacent NM plates [63].

## 4. Conclusions

(1) The influence of uniaxial compression on martensitic transformation in directionally solidified Ni_50_Mn_30_Ga_20_ and Ni_50_Mn_28.5_Ga_21.5_ polycrystalline alloys was studied by neutron diffraction. It was shown that the distribution of martensite variants can be tuned through cyclic thermo-mechanical treatments. For the Ni_50_Mn_30_Ga_20_ alloy with <0 0 1>_A_ preferential orientation parallel to the solidification direction, a strong <0 1 0>_7M_ preferential orientation of 7M martensite along the loading direction (//solidification direction) was induced by the external compression during martensitic transformation. In addition, it was found that the selection of preferential variants induced by thermo-mechanical treatments was strongly dependent on the austenite orientation and the direction of external load, which was evidenced in Ni_50_Mn_28.5_Ga_21.5_ polycrystalline alloys with <0 0 1>_A_ and <1 1 0>_A_ parallel to the solidification direction. For the austenite with the orientation of <0 0 1>_A_//SD, the compressive load applied along solidification direction favored the formation of variants with {1 0 5}_5M_/{−1 0 5}_5M_//SD (LD) and {0 2 0}_5M_⊥SD (LD). On the other hand, the formation of the variants with {2 0 0}_5M_/{0 0 10}_5M_//SD (//LD) and {1 2 5}_5M_/{−1 2 5}_5M_⊥SD (LD) was favored under the condition of LD//<1 1 0>_A_. The preferred orientation of the martensite variants formed under the external compression applied during the martensitic transformation should be attributed to the accommodation between the anisotropic lattice distortion in the martensitic transformation and the external constraint.
(2) The preferred orientation distribution for the austenite in Ni_53_Mn_22_Ga_25_ribbons and the 7M martensite in Ni_51_Mn_27_Ga_22_ ribbons was studied based on EBSD and XRD. It was found that the austenite forms a preferred orientation with {4 0 0}_A_ parallel to ribbon plane, whereas the 7M martensite develops the preferred orientation with {2 0 −20}_7M_, {2 0 20}_7M_, and {0 4 0}_7M_ crystallographic planes parallel to the ribbon plane. The preferred orientation distribution for austenite and 7M martensite was well correlated and the preferred orientation in ribbons can be inherited after the martensitic transformation. Such texture inheritance is attributed to the intrinsic orientation relationship between austenite and 7M martensite.
(3) Epitaxially grown thin films with nominal composition Ni_50_Mn_30_Ga_20_ and thickness of 1.5 μm were prepared on MgO(1 0 0) substrate with a Cr buffer layer by DC magnetron sputtering. Based on EBSD measurements, it was revealed that the coarse plates in the top layer of the film are of the NM martensite, whereas the fine plates in the film interior are of the 7M martensite. For both 7M and NM martensite, the plate-like microstructures are composed of two distinct kinds of plate groups with low or high relative contrast. For 7M martensite, {2 0 −20}_7M_, {2 0 20}_7M_ and {0 4 0}_7M_ are nearly parallel to the substrate surface, whereas for the NM martensite, {0 0 4}_NM_ and {2 2 0}_NM_ tends to be parallel to the substrate surface. EBSD measurements show that one plate group of 7M martensite consists of four twin-related variants. In the low relative contrast zone, the majority of variants are in Type-I twin relation, whereas for the high relative contrast zone, the majority of variants are in Type-II twin relation. The selection of twinning type is a consequence of external constraint from the rigid substrate and the twinning type with less shear deformation in the film normal direction is favored. For NM martensite, one plate group of NM martensite also consists of 4 martensite plates, but each plate is composed of two twin related variants with one thicker than the other. The in-plate major and minor variants are distributed symmetrically to the inter-plate interfaces in low relative contrast zones, but asymmetrically distributed in high relative contrast zones. The difference in the orientation combination of the in-plate variants accounts for the topological differences observed for the two relative contrast zones.

The presented investigations are expected to provide some fundamental information for the microstructure modification and functional performance control of ferromagnetic shape memory alloys.
